# Endomorphin-2 Decreases Excitatory Synaptic Transmission in the Spinal Ventral Horn of the Rat

**DOI:** 10.3389/fncir.2017.00055

**Published:** 2017-08-08

**Authors:** Zhen-Yu Wu, Ya-Cheng Lu, Ban Feng, Ying-Biao Chen, Yang Bai, Ting Zhang, Hua Zhang, Tao Chen, Yu-Ling Dong, Hui Li, Yun-Qing Li

**Affiliations:** ^1^Department of Anatomy and K. K. Leung Brain Research Centre, The Fourth Military Medical University Xi’an, China; ^2^Department of Anatomy, Fujian Health College Minhou, China; ^3^Department of Geriatrics, Xijing Hospital, The Fourth Military Medical University Xi’an, China; ^4^Collaborative Innovation Center for Brain Science, Fudan University Shanghai, China

**Keywords:** endomorphin-2, μ-opioid receptor, motor impairment, motoneuron, synapse, sEPSC, rat

## Abstract

Motor impairment is one of the serious side-effects of morphine, which is an exogenous agonist of the μ-opioid receptor (MOR) as well as a widely used analgesic drug in clinical practice for chronic pain treatment. Endomorphins (EMs, including EM-1 and EM-2), the most effective and specific endogenous agonists of the MOR, exert more potent analgesia in acute and neuropathic pain than other opiates, such as morphine. Although EMs had fewer side-effects comparing to other opiates, motor impairment was still one unwanted reaction which limited its clinical application. In order to prevent and treat the motor impairment, it is critical to reveal the neural mechanisms underlying such locomotion disorder. The purpose of the present study was to reveal the neural mechanisms underlying the effects of EM-2 on the activity of motoneurons in the spinal ventral horn. First, we examine the distribution of EM-2-immunoreactive (IR) primary afferent fibers and their synaptic connections with the motoneurons innervating the skeletal muscles of the lower limb revealed by sciatic nerve retrograde tracing. The results showed that EM-2-IR fibers and terminals were sparsely observed in lamina IX and they formed symmetric synaptic connections with the motoneurons within lamina IX of the spinal ventral horn. Then, whole-cell patch-clamp technique was used to observe the effects of EM-2 on the spontaneous excitatory postsynaptic current (sEPSC) of motoneurons in lamina IX. The results showed that EM-2 could decrease both the frequency and amplitude of the sEPSC of the motoneurons in lamina IX, which was reversed by the MOR antagonist CTOP. These results indicate that EM-2-IR fibers originated from primary afferent fibers form symmetric synaptic connections with motoneurons innervating skeletal muscles of the lower limbs in lamina IX of the spinal ventral horn and EM-2 might exert inhibitory effects on the activities of these motoneurons through both presynaptic and postsynaptic mechanisms.

## Introduction

Morphine is the most effective analgesic in clinical treatment, especially in postoperative pain and neuropathic pain (Corbett et al., 2006). During the application of the morphine, there are many side-effects, such as tolerance, addiction, respiratory depression, constipation, urinary retention, and motor impairment, which interfere with its clinical usage(Fiserova and Metys, 1989; Czapla et al., 2000; Walters et al., 2005; Ruan, 2007; Khansari et al., 2013; Yan et al., 2014; Trang et al., 2015; Zadina et al., 2016). The neural mechanisms for the side-effects of morphine have been investigated systemically, except for the motor impairment (Walters et al., 2005; Zadina et al., 2016). Motoneurons in spinal ventral horn are essential for the locomotion of the body and limbs and play important roles in both somatic movements and motor disorders (Blumenfeld, 2010). Patients suffering from long-term movement disorders exhibited obvious neuronal loss and gliosis in spinal ventral horn (Kaneto et al., 2000).

μ-Opioid receptor (MOR), one major specific receptor for morphine, is expressed in the motoneurons within the spinal ventral horn (Gouarderes et al., 1991; Kar and Quirion, 1995; Mansour et al., 1995). Endomorphins (EMs, including EM-1 and EM-2 subtypes), the most effective and specific endogenous agonists of MOR, have vast potential usage for future development in analgesia (Zadina et al., 1997, 2016). However, the undesirable effects of EMs emerged in certain animal experiments, such as tolerance, addiction, and motor impairment, still restricted their therapeutic development as analgesics (Hung et al., 2002; Labuz et al., 2002; Chen et al., 2003). EM-1 and EM-2, especially EM-2, could modulate neuronal activities in the spinal dorsal horn through activating both pre- and postsynaptically located MORs (Sakurada et al., 1999; Zadina et al., 1999; Smith et al., 2001; Wu et al., 2003; Leng et al., 2005; Fichna et al., 2007). Previous studies have shown that EM-2-immunoreactive (IR) fibers and terminals were sparsely distributed in the spinal ventral horn (Martin-Schild et al., 1999). Tyr-D-Ala-Gly-N-Met-Phe-Gly-ol (DAMGO), an artificially synthesized agonist of the MOR, hyperpolarizes lamina IX motoneurons in the spinal ventral horn by G protein-mediated activation of potassium channels after activation of MORs. Furthermore, DAMGO decreased the frequency of spontaneous excitatory and inhibitory postsynaptic currents to reduce both excitatory and inhibitory transmitter release. However, they just recorded lamina IX neurons (not identified spinal motoneurons), and failed to exclude some spinal interneurons (Honda et al., 2012). All of these results suggested that EM-2 might also play a crucial role in locomotion regulation at the spinal cord level. In order to prevent and treat EM-2 induced motor disorders, it is fundamental to identify the effects of EM-2 on spinal motoneurons and explore the related neural mechanisms. Thus, the present study aimed to observe EM-2’s regulatory effects on the activity of spinal motoneurons (identified by sciatic nerve retrograde labeling) in the spinal ventral horn by whole cell patch-clamp recording. Immunoelectron microscope technique was to show the synaptic connections between EM-2 terminals and motoneurons, providing direct morphological evidence for these electrophysiological results.

## Materials and Methods

### Experimental Animals

All the animals were conducted using approved protocols and carried out in accordance with the principles of Laboratory Animal care (NIH Publication No. 85-23, revised 1985). The experimental procedures in this study were approved by The Committee of Animal Use for Research and Education in The Fourth Military Medical University (Xi’an, China). Eleven adult male Sprague-Dawley rats (weighing 200–250 g) were used for morphological study. Seventeen Sprague-Dawley rats at the age of postnatal day 7–10 (weighing 25–30 g) were adopted for retrograde tracing and then used for whole-cell patch-clamp recording on the spinal cord slice 3 days later. All efforts were made to minimize animal suffering as well as the number of animals used.

### Retrograde Tracing

Eleven rats were deeply anesthetized with intraperitoneal injection of sodium pentobarbital (45 mg/kg) dissolved in 0.9 % (*w*/*v*) saline. The left sciatic nerve was exposed for retrograde labeling of motoneurons with tetramethylrhodamine dextran amine (TMR-DA, D3308, 3000 MW, Molecular Probes, Eugene, OR, United States) (six rats) or wheat germ agglutinin-conjugated horseradish peroxidase (WGA-HRP, Toyobo, Tokyo, Japan) (five rats). When using TMR-DA as the tracer, we repeated painting an approximate volume of 1 μl of a 10% TMR-DA dissolved in 0.1 M citrate-NaOH (pH 3.0) on sciatic nerve (Kaneko et al., 1996; Li et al., 1997). When using WGA-HRP as the tracer, we injected a solution containing 2% WGA-HRP into the nerve (Dou et al., 2013).

### Immunohistochemistry

Three days after TMR-DA injection, six rats were deeply anesthetized with sodium pentobarbital (100 mg/kg, i.p.) and transcardially perfused with 100 ml of 0.05 M phosphate-buffered saline (PBS, pH 7.4), followed by a volume of 500 ml of 0.1 M phosphate buffer (PB) containing 4% paraformaldehyde and 30% (v/v)-saturated picric acid. Lumbar spinal cord were removed and cryoprotected in 0.1 M PB containing 30% (w/v) sucrose at 4°C for 24 h. Subsequently, 30-μm transverse sections were cut using a cryostat microtome (CM1950, Leica) and collected into 0.01 M PBS (pH 7.4).

The intact sections were chose for TMR and EM-2 or MOR double staining. The sections were blocked with 10% normal goat serum in 0.01 M PBS (pH 7.4) and then incubated for 24 h with guinea pig anti-TMR (1:500, donated from professor Takeshi Kaneko, Kyoto University; Kaneko et al., 1996) and rabbit anti-EM-2 (1:200; AB10289, Abcam, Cambridge, MA, United States) or rabbit anti-MOR (1:500; AB10275, Abcam) antibodies at room temperature (RT). After washing in 0.01 M PBS (pH 7.4), sections were incubated for 8 h at RT with: (1) biotinylated donkey anti-rabbit IgG (1:500; Millipore, AP182F, Billerica, MA, United States) and Alexa594-conjugated donkey anti-guinea pig IgG (1:500; 706-585-148, Jackson ImmunoResearch, Suffolk, United Kingdom) diluted with PBS-NDS (for TMR and EM-2 staining); (2) biotinylated donkey anti-rabbit IgG (1:500; AP182F, Millipore, Billerica, MA, United States) and Alexa488-conjugated donkey anti-guinea pig IgG (1:500; 706-545-148, Jackson ImmunoResearch, West Grove, PA, United States) diluted with PBS-NDS (for TMR and MOR staining). Then, after washing in 0.01 M PBS (pH 7.4), sections were incubated with FITC-Avidin (1:1,000; A-2001, Vector, Burlingame, CA, United States) (for EM-2 staining) or Alexa594-Avidin (1:1,000; S32356, Invitrogen, Carlsbad, CA, United States) (for MOR staining) in 0.01 M PBS containing 0.5% Triton X-100 (PBS-X) for 3 h at RT. Finally, the sections were rinsed with 0.01 M PBS, mounted onto clean glass slides, air-dried and cover slipped with a mixture of 0.05 M PBS containing 50% (v/v) glycerin and 2.5% (w/v) triethylenediamine. Images were taken using a laser-scanning confocal microscope (FV1000, Olympus, Tokyo, Japan).

### Electron Microscopy

After surviving for 3 days, all the rats retrograde labeled by WGA-HRP were deeply anesthetized by injection of an overdose of sodium pentobarbital (100 mg/kg, i.p.), and then perfused with 100 ml of 0.01 M PBS (pH 7.4), followed by 500 ml of a fixative consisting of 4% paraformaldehyde and 0.01% glutaraldehyde in 0.1 M PB (pH 7.4). The spinal cord were removed and stored in 0.1 M PB (pH 7.4) containing of 4% paraformaldehyde at 4°C for 2–4 h.

Serial sections of the lumbar enlargement were cut transversely on a vibratome (DTK-1000, Dosaka, Kyoto, Japan) at 50 μm thickness. Tissue sections were processed for the histochemical demonstration of WGA-HRP by using tetramethylbenzidine-sodium tungstate (TMB-ST) method and the WGA-HRP reaction products were intensified with a 3,3-diaminobenzidine tetrahydrochloride (DAB)/cobalt/H_2_O_2_ solution (Gu et al., 1991). The sections were then mounted on to glass slides, and the distribution of WGA-HRP-labeled neurons in the anterior horn of the spinal cord was examined using a light microscope. Sections containing WGA-HRP-labeled neurons were selected for further study. These sections were cryoprotected in 10, 20, and 30% sucrose in 0.05 M PB that contained 10% (v/v) glycerol for 0.5 h, and then freeze-thawed with liquid nitrogen to enhance the degree of antibody penetration. After 0.5 h of blocking with 10% FCS in PBS (0.01 M, pH 7.4), immunoperoxidase methods were used to label EM-2. The sections were then incubated in 20% normal goat serum (Vector) in 50 mM Tris-buffered saline (TBS, pH 7.4) for 0.5 h to block the non-specific immunoreactivity. The sections were then incubated with a mixture of rabbit anti-EM-2 (1:100; AB10289, Abcam) in TBS containing 2% (v/v) normal donkey serum (TBS-D) for 72 h, washed in 0.05 M TBS and subjected to an overnight incubation with a mixture of 1:500-diluted biotinylated anti-rabbit IgG (AP182F, Millipore) in TBS-D. After rinsing three times in TBS, the sections were placed in a 0.05 M Tris–HCl (pH 7.5) solution containing 0.02% DAB (Dojin, Kumamoto, Japan) and 0.003% H_2_O_2_ for about 0.5 h. The immunolabeled sections were then postfixed in 1% OsO_4_, counterstained with 1% uranyl acetate in 70% ethanol, dehydrated, flat-embedded in Durcupan (Fluka, Buchs, Switzerland) and polymerized. Small pieces of the anterior horn of the spinal cord that contained a large number of WGA-HRP retrogradely labeled neuronal cell bodies and axon terminals were selected and removed from the flat-embedded sections under a dissection microscope. The selected tissue pieces were then cut into serial, ultrathin sections with an ultratome (Reichert-Nissei Ultracut S, Leica, Wien, Austria), and then mounted on to single-slot grids that had been coated with pioloform membrane, stained with 1% lead citrate, and finally examined with an electron microscope (CM100, Philips, Eindhoven, Netherlands).

### Spinal Cord Slice Preparation

Male Sprague-Dawley rats aged 7–10 days were retrograde traced by TMR-DA as previously described. After surviving for 3 days, they were deeply anesthetized by sodium pentobarbital (100 mg/kg, i.p.). The spinal cord at L1–L5 was removed using pressurized 2–4°C sucrose-aCSF with a 2-ml syringe. The sucrose-aCSF consisted of the following reagents (in mM): 220 sucrose, 2.5 KCl, 26 NaHCO_3_, 6.0 MgSO_4_, 1.2 KH_2_PO_4_, 0.5 CaCl_2_, 10 glucose, 1 ascorbate, and 3 sodium pyruvate. The lumbar segment was sectioned to 400 μm thickness with a vibratome (Leica VT 1200s, Heidelberger, Nussloch, Germany) in 2–4°C sucrose-aCSF bubbled with 95% O_2_ and 5% CO_2_. The spinal slices were transferred to an incubation chamber filled with normal-sodium aCSF (normal-aCSF) that was continuously bubbled with 95% O_2_ and 5% CO_2_ and incubated at RT (22–24°C) until use. The normal-aCSF contained (mm): 124 NaCl, 2.5 KCl, 25 NaHCO_3_, 2 MgSO_4_, 1 NaH_2_PO_4_, 2 CaCl_2_, 10 glucose, 1 ascorbate, and 3 sodium pyruvate.

### Whole Cell Patch-Clamp Recording

After a 1 h recovery period, the slices were transferred to a glass-bottomed recording chamber which had a volume of 0.5 ml, and fixed with parallel nylon threads supported by a U-shaped stainless steel weight. The slices were continuously perfused with normal-aCSF bubbled with 95% O_2_ and 5% CO_2_ at a rate of 2-3 ml/min. The experiments were performed at 30 ± 1°C using a heat controller. Motoneurons in lamina IX of the spinal ventral horn were identified with differential interference contrast/infrared illumination on a fixed-stage microscope (BX51W1 Olympus, Tokyo, Japan). Whole cell patch-clamp recordings were performed on TMR-labeled motoneurons in the lamina IX that were visualized under epifluorescence using a tetramethylrhodamine isothiocyanate (TRITC) filter set (U-HGLGPS, Olympus) with a monochrome CCD camera (IR-1000E, DAGE-MTI, Michigan, United States) and monitor. The patch pipettes were constructed with a P-97 micropipette puller (Sutter Instruments, Novato, CA, United States) from borosilicate glass capillary tubes (World Precision Instruments, Sarasota, FL, United States). The tip resistance was in the range of 3–5 MΩ when filled with the internal pipette solution which containing: 130 potassium gluconate, 15 KCl, 5 NaCl, 10 4-(2-hydroxyethyl)-1-piperazineethanesulfonic acid (HEPES), 4 Mg-ATP, 0.3 Na_2_-GTP, and 0.4 ethylene glycol tetraacetic acid (EGTA) (pH 7.3; osmolarity, 290–300 mOsm/L), and 0.05% biocytin (Sigma-Aldrich, St. Louis, MO, United States) was added in all of the experiments to visualize the recorded neurons. The neurons were recorded using a Multiclamp 700B amplifier (Axon Instruments, Foster City, CA, United States). The pCLAMP software (v.10.02, Axon Instruments) was used to acquire and analyze the data. The signals were filtered at 2.6 kHz, digitized at 10 kHz (Digidata 1322A, Axon Instruments), and saved on a computer for offline analysis. Recordings that met the following criteria were included in the analyses: a resting membrane potential of at least -45 mV (the liquid junction potential was not corrected and was 13.7 mV in our experimental conditions), and series resistance (Rs) was ≤20 MΩ. Average Rs did not change by more than 10% in any of the accepted recordings.

The avidin–biotin complex reaction with a fluorescent labeling was used to visualize the biocytin-labeled cell bodies of motoneurons. After recording, the slices were fixed in 4% paraformaldehyde in 0.1 M PB (pH 7.4) for 4 h and were subsequently stored in 30% sucrose solution overnight at 4°C. After three rinses with 0.01 M PBS, the sections were blocked with 10% normal goat serum in 0.01 M PBS (pH 7.4) and incubated for 24 h with FITC-avidin (1:1,000; Vector Laboratories, A-2001, CA, United States) in 0.01 M PBS (pH 7.4). The slices were mounted, cover-slipped and examined using a confocal laser-scanning microscope (FV1000, Olympus).

### Statistical Analysis

All numerical data were expressed as the means ± the SD. For the electrophysiology data, *n* refers to the number of neurons studied. EM-2 was bath-applied for about 8 min and then washed out in each recording. The cumulative probabilities of the inter-event intervals and the amplitudes of the spontaneous excitatory postsynaptic currents (sEPSCs) were compared using Kolmogorov–Smirnov tests using the 3 min duration before drug application as the control amplitude or frequency for comparison with the amplitudes or frequencies of 3 min duration after 5 min of drug application. Group means were compared using paired *t*-tests for paired data. Differences between the means were considered significant at *P* < 0.01.

## Results

### Connections between EM-2-IR Terminals and Motoneurons in the Spinal Ventral Horn

In order to examine the distribution of EM-2-like IR fibers and terminals and the connections between EM-2-IR fibers and motoneurons in lamina IX of the spinal ventral horn, a double-labeling method combining TMR-DA retrograde tracing with immunofluorescence histochemical staining for EM-2 was applied.

**FIGURE 1:**
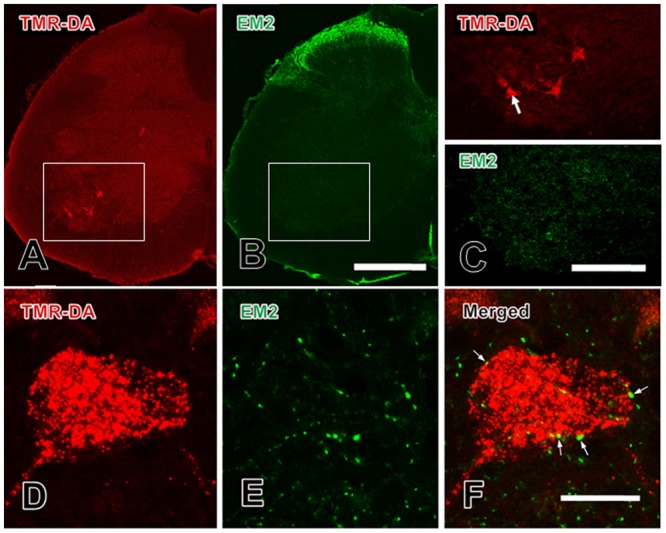
Close contacts between EM-2-IR terminals and TMR-DA retrogradely labeled motoneurons in the spinal ventral horn. **(A)** TMR-DA labeled motoneurons in lamina IX; **(B)** EM-2-IR fibers and terminals in spinal dorsal and ventral horns; **(C)** the areas indicated with white rectangles in **(A)** and **(B)** was enlarged in **(C)** to show the morphological features of the TMR-DA labeled motoneurons and EM-2-IR fibers and terminals in lamina IX; **(D–F)** the close contacts (indicated with small white arrows) between EM-2-IR fibers and terminals and a TMR-DA labeled motoneuron indicated with a large white arrow in **(C)**. Scale bars = 280 μm in **(A)** and **(B)**, 140 μm in **(C)**, and 20 μm in **(D–F)**.

After injection of TMR-DA into the peripheral portion of the sciatic nerve at the middle of the thigh, some large neuronal cell bodies within lamina IX innervating the skeletal muscles of the lower limb through sciatic nerve were labeled by TMR-DA (**Figures 1A,C**). These TMR-DA labeled neurons were in multipolar shape. Their cell bodies and large dendritic processes were filled with red granular particles, which were enhanced by immunofluorescent histochemical staining with the TMR-DA antibody (**Figures 1D,F**).

The results of immunofluorescent histochemical staining for EM-2 showed that EM-2-IR fibers and terminals were densely found in the superficial laminae (lamina I and lamina II) of the spinal dorsal horn (**Figure 1B**), whereas EM-2-IR fibers and terminals were sparsely encountered in lamina IX of the spinal ventral horn (**Figure 1E**). There were no EM-2-IR cell bodies identified in either spinal dorsal or ventral horns (**Figure 1**). Some EM-2-IR fibers and terminals made close contacts with the outer-membrane surface of the TMR-DA-labeled neuronal cell bodies and their dendritic processes (**Figures 1D–F**).

### Synaptic Connections between EM-2-IR Terminals and Motoneurons in the Spinal Ventral Horn

In order to provide convincing morphological evidence for these connections between EM-2-IR fibers and motoneurons in lamina IX of the spinal ventral horn observed under light microscope, electron microscopy was performed subsequently to demonstrate the synaptic connections between them by WGA-HRP retrograde tracing combined with immunohistochemical staining for EM-2.

**FIGURE 2:**
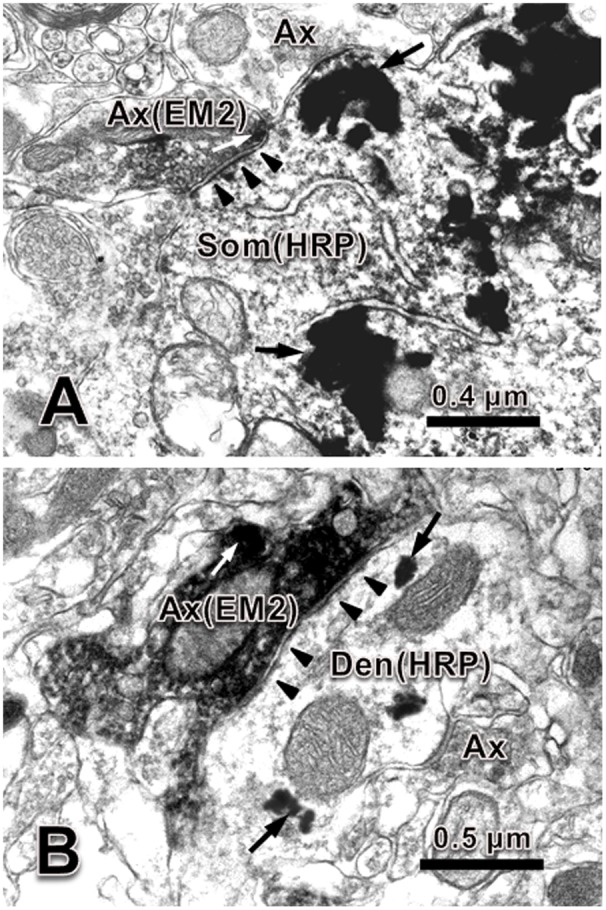
Synaptic connection between EM-2-IR terminal and HRP labeled dendrite and soma of motoneuron in spinal ventral horn. **(A)** An EM-2-IR axon terminal (Ax) contained EM-2-immunopositive large dense coated vesicle (white arrow, labeled with DAB reaction products) made symmetric synapse with an HRP (black arrows, labeled with TMB-ST reaction products) labeled soma (Som). **(B)** An EM-2-IR axon terminal contained several EM-2-immunopositive large dense coated vesicle (white arrow, labeled with DAB reaction products) made symmetric synapse with an HRP (black arrows, labeled with TMB-ST reaction products) labeled dendrite (Den). Black arrowheads indicate the postsynaptic membranes. Scale bars = 0.4 μm in **(A)**, 0.5 μm in **(B)**.

Under the electron microscope, EM-2-IR axonal terminals within lamina IX of the spinal ventral horn were characterized by the presence of electron dense DAB reaction products adhering to the outer surface of cell organelles such as mitochondria and synaptic vesicles and the inner surface of the plasma membrane. EM-2-IR terminals were usually filled with several large dense coated vesicles (LDCVs), which are believed to contain neuropeptides (**Figure 2**). HRP retrogradely labeled motoneurons were determined by the presence of the TMB-ST reaction products distributed in the cytoplasm and dendrites of the motoneuron enhanced by the DAB and heavy metals. These reaction products tended to be localized homogenously in both the cytoplasm and the processes of the neuronal cell bodies (**Figure 2**) of the HRP labeled motoneurons. The synaptic connections between EM-2-IR terminals and HRP labeled somatic profiles and dendritic processes of the motoneurons were observed, the overwhelming majority of which were symmetric synapses (**Figure 2**).

### Expression of MOR on Motoneurons in the Spinal Ventral Horn

To show if MOR was expressed on motoneurons in lamina IX of the spinal ventral horn, a double-labeling method by combining TMR-DA retrograde tracing with immunofluorescence histochemical staining for MOR was applied.

**FIGURE 3:**
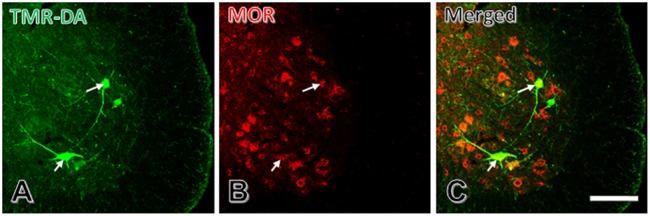
Some spinal ventral horn TMR-DA retrogradely labeled motoneurons are MOR positive. **(A)** TMR-DA labeled motoneurons in spinal ventral horn. **(B)** MOR positive motoneurons in spinal ventral horn. **(C)** Merged image of **(A)** and **(B)**. White arrows indicate two TMR-DA and MOR double-labeled neurons. Scale bar = 90 μm.

After injection of TMR-DA into the peripheral portion of the sciatic nerve, cell bodies of some large neurons cell bodies and large dendritic processes were labeled by TMR-DA (**Figure 3A**). The results of immunofluorescent histochemical staining for MOR showed that there were many MOR positive neurons in the spinal ventral horn (**Figure 3B**). About 70% TMR-DA-labeled neurons expressed MOR (**Figure 3C**).

### Modulation Effects of EM-2 on the Activity of Motoneurons in the Spinal Ventral Horn

In order to reveal the modulatory effects of EM-2 on motoneurons in the spinal ventral horn, whole-cell patch-clamp recording combined with TMR-DA retrograde tracing technique was used.

**FIGURE 4:**
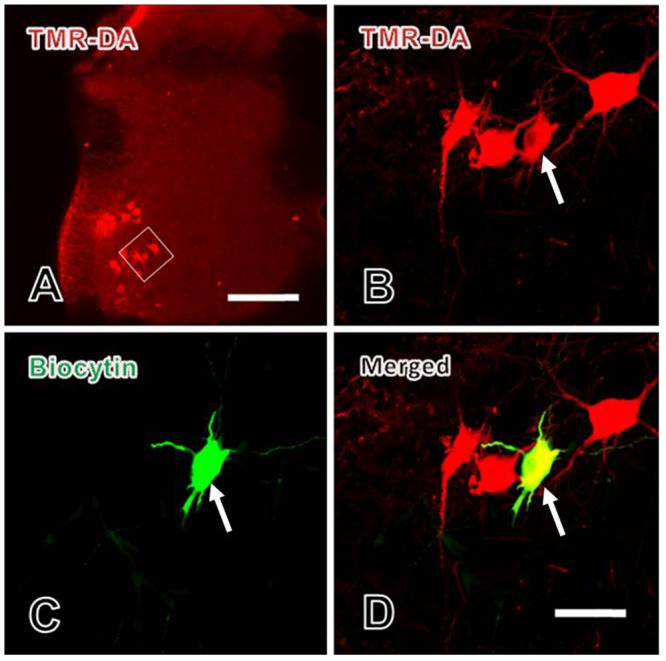
Verification of the patch-clamp recorded neurons in spinal ventral horn. **(A)** The lower power image of TMR-DA labeled motoneuron (red) in lamina IX of spinal ventral horn in which TMR-DA was injected into the sciatic nerve. The white rectangle in **(A)** was magnified in **(B)**. **(B–D)** The higher power images of the TMR-DA labeled motoneuron (red in **B**) also showed biocytin positive labeling (green in **C**), i.e., the TMR-DA/biocytin double labeled motoneuron (yellow in **D**, indicated with white arrows) in lamina IX of the spinal ventral horn. Scale bars = 200 μm in **(A)**, 25 μm in **(B–D)**.

Three days after TMR-DA injection into the sciatic nerve of rat, the motoneurons in lamina IX were recorded. Before recording, TMR-DA retrogradely labeled motoneurons in lamina IX were selected under fluorescence microscope. During recording, biocytin was microiontophoretically injected into the recorded motoneurons by the recording microelectrode. After recording, immunofluorescent histochemical staining for biocytin was performed to verify that every recorded neuron was TMR-DA labeled motoneuron (**Figure 4**).

sEPSCs were recorded using the whole-cell voltage-clamp recording method as described previously (Chen et al., 2015). Whole-cell voltage-clamp recordings (clamped at -70 mV) were made from TMR-DA labeled motoneurons in lamina IX, and high-pass and low-pass filters were selected to eliminate the baseline fluctuations and noise. To examine the effects of EM-2 on the sEPSCs of these motoneurons, 100 μM picrotoxin and 2 μM strychnine were applied to eliminate the influence of inhibitory neurotransmission. At the end of the experiments, the sEPSCs were blocked by bath application of the 10 μM α-amino-3-hydroxy-5-methyl-4-isoxazolepropionic acid (AMPA) receptor antagonist 6-cyano-7-nitroquinoxaline-2,3-dione (CNQX), which confirmed that the sEPSCs were glutamate-mediated postsynaptic current (*n* = 3, data not shown).

### EM-2 Decreased the Amplitude and Frequency of sEPSCs

**FIGURE 5:**
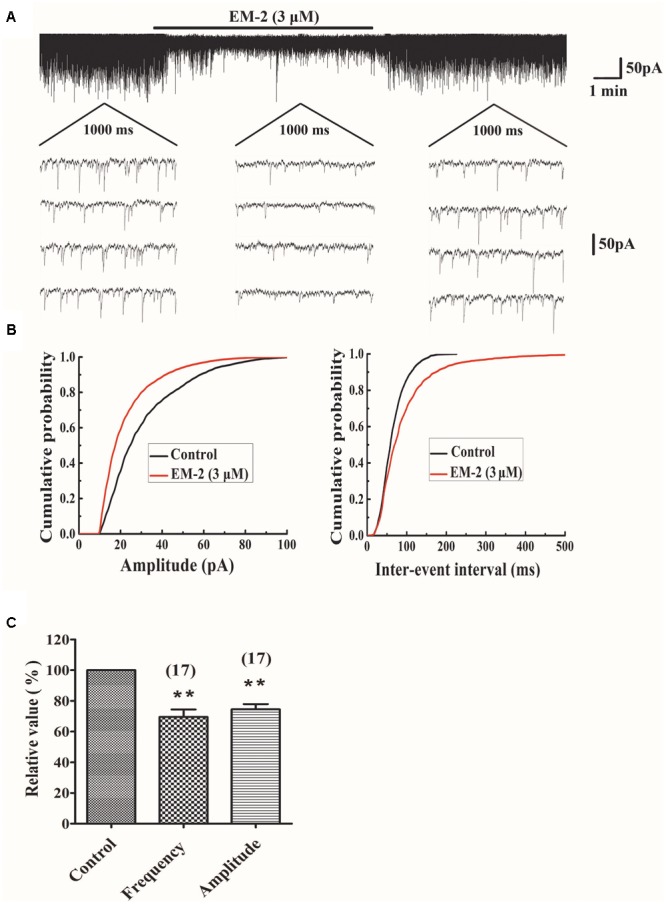
EM-2 decreased amplitudes and frequencies of sEPSCs in TMR-DA labeled ventral horn motoneurons. **(A)** Sample traces of sEPSCs in the absence and presence of EM-2. In this and subsequent figures, the duration of drug superfusion is shown by a horizontal bar above the chart recording, and four consecutive traces of sEPSCs are shown in the expanded time scales. The period of each trace is 1,000 ms. **(B)** Cumulative distributions of the amplitudes (left) and inter-event intervals (right) of the sEPSC before (black line) and during (red line) the EM-2 applications. EM-2 significantly shifted the mean cumulative distribution of the sEPSC amplitude to lower amplitudes (*P* < 0.01; Kolmogorov–Smirnov test) and significantly shifted the mean cumulative distribution of the sEPSC inter-event intervals to longer durations (*P* < 0.01; Kolmogorov–Smirnov test). The data were obtained from the same neuron as in panel **(A)**. **(C)** Histogram showing that the sEPSC amplitude and frequency were significantly reduced by EM-2 (*P* < 0.05, paired *t*-test *n* = 17). In this and the following figures, the numbers of neurons used to construct each data point are shown in parentheses. The vertical bars show the SDs. ^∗∗^*P* < 0.01 versus control. Holding potential = –70 mV.

The effects of EM-2 on sEPSCs were studied in 17 TMR-DA labeled motoneurons in lamina IX of the ventral horn. In 11 of 17 tested motoneurons, perfusion of EM-2 (3 μM) for 8 min resulted in a reversible reduction in amplitude of sEPSCs (**Figure 5A**). The remaining six motoneurons showed a non-significant change in sEPSC amplitude. Overall sEPSC amplitude was reduced from -33.9 ± 21.5 to -23.5 ± 13.6 pA after EM-2 perfusion (74.5 ± 17.8% of the control sEPSC amplitude, *P* < 0.01; paired *t*-test *n* = 17, **Figures 5B,C**). Meanwhile, the average input resistance of the 11 positively responding neurons decreased from 470.6 ± 220.5 to 369.5 ± 189.6 MΩ (*P* < 0.01; paired *t*-test, *n* = 11). The sEPSC frequency was also significantly reduced in 16 of 17 tested ventral horn neurons and increased in one tested neuron. Overall, sEPSC frequency was reduced from 4.38 ± 2.5 to 3.07 ± 2.12 Hz after EM-2 perfusion (69.5 ± 20.3% of the control sEPSC frequency; *P* < 0.01; paired *t*-test, *n* = 17, **Figures 5B,C**).

### CTOP Blocked the Action of EM-2 on sEPSCs

**FIGURE 6:**
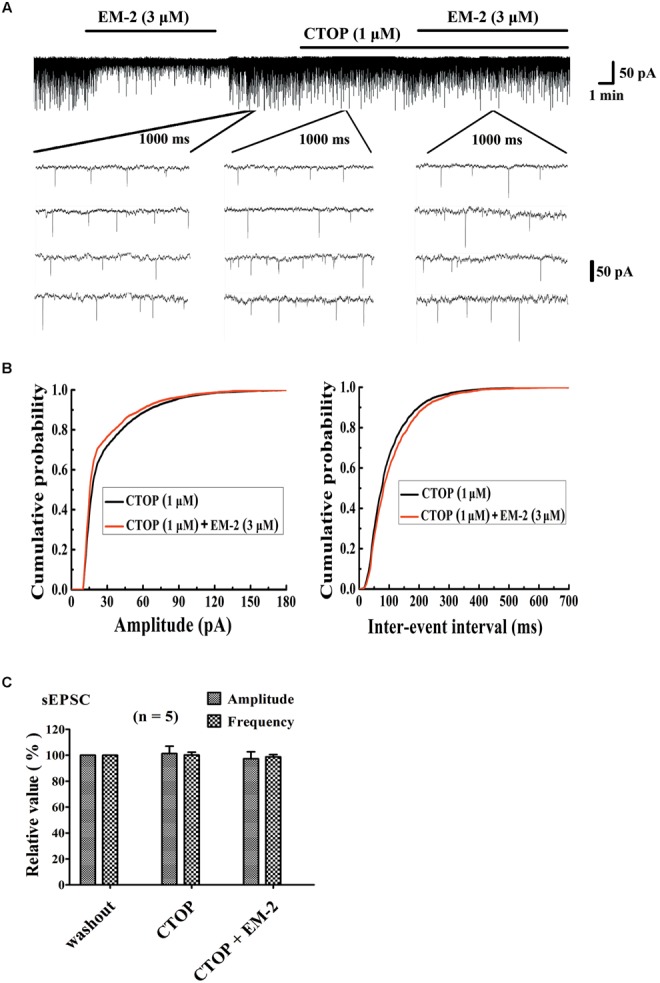
CTOP blocked the action of EM-2 on sEPSCs in motoneurons. **(A)** A sample trace of sEPSCs in the presence and wash-out of EM-2 (3 μM), and subsequently the presence of CTOP (1 μM) and EM-2 (3 μM) together with CTOP (1 μM). **(B)** Cumulative distributions of the amplitudes (left) and inter-event intervals (right) of the sEPSCs in the presence of CTOP (black line) and EM-2 together with CTOP (red line). EM-2 did not significantly shift the mean cumulative distribution of the sEPSC amplitude or the inter-event intervals (*P* > 0.05; Kolmogorov–Smirnov test). These data were obtained from the same neuron as in panel **(A)**. **(C)** Bar graph show the analytical results of three continuous recordings during which the wash-out of EM-2, presence of CTOP and EM-2 together with CTOP were treated in five ventral horn neurons. CTOP had no effect on either the amplitudes or frequency of sEPSCs (*P* > 0.05), with perfusion of both CTOP and EM-2, EM-2 was unable to decrease the amplitudes or frequency of the sEPSCs (*P* > 0.05). Holding potential = –70 mV.

To investigate whether the inhibitory effects of EM-2 on lamina IX motoneurons within the spinal ventral horn were mediated by the MORs, the selective MOR antagonist D-Phe-Cys-Tyr-D-Trp-Orn-Thr-Pen-Thr-NH2 (CTOP, 1 μM) were used to block MORs in the electrophysical recordings. We examined five motoneurons in the ventral horn, the activity of which were inhibited by EM-2 (**Figure 6A**). After 5–8 min of washout of the EM-2, the amplitudes and frequencies returned to the basal levels. Bath application of CTOP (1 μM) had no effect on the amplitudes or frequencies of the sEPSCs (101.3 ± 5.7% of the wash out sEPSC amplitude and 100.2 ± 2.2% of the wash out sEPSC frequency; *n* = 5; *P* > 0.05). However, perfusion with EM-2 after pretreatment with CTOP had no effect on the sEPSC amplitudes and frequencies (97.3 ± 5.4% of the wash-out sEPSC amplitude and 98.7 ± 1.8% of the wash-out sEPSC frequency; *n* = 5; *P* > 0.05; **Figures 6B,C**). These data suggested that the inhibitory effects of EM-2 on the activity of motoneurons within the ventral horn were mediated by the MORs.

## Discussion

EM-1 and EM-2 are two endogenous ligands in the opioid peptide family, first discovered and isolated by Zadina and his colleagues in 1997 (Zadina et al., 1997, 1999; Aicher et al., 2003; Wang et al., 2003). Both EM-1 and EM-2 have the highest affinity for the MOR of all known mammalian opioids (Zadina et al., 1997, 1999). EM-1-IR and EM-2-IR fibers and terminals are usually prominently present in MORs-concentrated regions. However, there are negligible EM-1-IR and EM-2-IR structures in the striatum, a region known to express high levels of MORs (Ding et al., 1996; Martin-Schild et al., 1997, 1999; Zadina et al., 1999; Pierce and Wessendorf, 2000; Zadina, 2002). Thus, it is likely that although EM-1 and EM-2 are specific MOR ligands, they might be not the exclusive ligands for the MORs in the central nervous system.

Morphine, an exogenous agonist of MORs, has clear analgesic efficacy in clinical treatment. However, the side-effects, such as drug addiction, tolerance, and motor disorders, have limited its clinical applications. Many previous studies have addressed the neural mechanisms for those side-effects except for motor impairment (Fiserova and Metys, 1989; Czapla et al., 2000; Walters et al., 2005; Ruan, 2007; Khansari et al., 2013; Yan et al., 2014; Trang et al., 2015; Zadina et al., 2016).

Opioids mainly exert inhibitory effects in the central nervous system, leading to a reduction in postsynaptic neuronal excitability or presynaptic excitatory neurotransmitter release. The results of previous researches suggested that opioids could modulate the excitability of spinal motoneurons (Fernandez-Galinski et al., 1996; Thees et al., 1999; Scheufler and Zentner, 2002). Via binding to MOR, opioids decreased the amplitude of motor-evoked potentials and increased the latency (Fernandez-Galinski et al., 1996). Later, Honda et al. (2012) examined the modulatory effects of MOR agonist Tyr-D-Ala-Gly-N-Met-Phe-Gly-ol (DAMGO) on the motoneurons in the spinal ventral horn of rats by whole-cell patch-clamp recording technique. It was observed that DAMGO hyperpolarized spinal motoneurons by G protein-mediated activation of K^+^ channels, and reduced both the excitatory and inhibitory transmitter release (Honda et al., 2012).

EM, an endogenous ligand of MOR, possesses morphine-like regulatory effects but has less side-effects than traditional opioid analgesics. Animal studies on intrathecal EM injection suggested that high dose of EM induced motor impairment (Horvath et al., 1999). Therefore, it is necessary to observe if EM fibers directly contact with spinal motoneurons, and explore the effects of EM on it. It will be helpful for treating EM induced motor disorders. The results of previous studies have demonstrated that EM-1-IR structures were widely and densely distributed throughout the brain and upper brainstem (Martin-Schild et al., 1999). However, EM-2-IR fibers and terminals were mainly observed in lamina II, also called substantia gelatinosa (SG) of the medulla and the spinal cord dorsal horn (Martin-Schild et al., 1999; Pierce and Wessendorf, 2000), and sparsely distributed in the spinal ventral horn (Martin-Schild et al., 1999). In our previous study, we have demonstrated that EM-2-IR fibers and terminals in the spinal dorsal horn might originate from primary afferent fibers, intrinsic local neurons, and descending projections from the brainstem (Hui et al., 2010; Zhu et al., 2011). Considering this, we chose EM-2 to explore its regulatory effects on the motoneuronal activity in the present study.

We observed that EM-2 had two kinds of effects on sEPSC of ventral horn motoneurons. Recordings of glutamatergic sEPSCs may consist of a mixture of presynaptic action potential-dependent transmitter release and random quantal transmitter release (Scanziani et al., 1992). In most of the motoneurons recorded in the present study, both frequency and amplitude of sEPSC decreased after applying EM-2, and only about 22% recorded motoneurons had no changes. The decrease in the amplitude of the sEPSC might depend on MORs expression in the recorded motoneurons as previous studies showed that just a part of motoneurons in spinal ventral horn express MOR (Gouarderes et al., 1991; Kar and Quirion, 1995; Mansour et al., 1995). By using retrograde tracing, we were able to verify that recorded neurons belonged to motoneurons in lamina IX of the spinal ventral horn, and we also observed about 70% of them expressed MOR. Meanwhile, 11 of 17 tested motoneurons showed a decrease in the input resistance as well as reduced sEPSCs upon EM-2 perfusion. The decrease in the input resistance was consistent with a previous study reported by Honda et al. (2012) that DAMGO caused outward currents in ventral horn motoneurons. These results suggested that most of ventral horn motoneurons expressed MOR, and EM-2 could exert its inhibitory effects on motoneurons via acting on MOR. However, the physiological significance of endogenous release of EM-2 on the motoneurons is not clear and need to be further investigated.

Retrograde tracing combined with immunofluorescent histochemical staining or immunoelectron microscopy techniques were used to verify the existence of synaptic connections between EM-2-IR fibers and motoneurons in lamina IX. In line with the inhibitory effects of EM-2 on motoneurons, the synaptic connections between EM-2 terminals and the soma or dendrites of motoneurons were all symmetric synapses (inhibitory synapses).

The inhibitory effects of opioids primarily involve presynaptic inhibition of excitatory neurotransmitter release and postsynaptic hyperpolarization through the opening of K^+^ channels, which causes a reduction in neuronal excitability (North, 1992; Williams et al., 2001). In the present study, it was noticed that both the frequency and amplitude of the sEPSCs of spinal ventral horn motoneurons were decreased, indicating that both postsynaptic and presynaptic MORs participated in the inhibitory effects of EM-2. The present results seemed different from a previous report suggesting that DAMGO hyperpolarized spinal lamina IX neurons by G protein-mediated activation of K^+^ channels after activation of MORs, and activation of MORs on presynaptic terminals reduced both excitatory and inhibitory transmitter release, and they only reported a decrease in sEPSC frequency, but not amplitude (Honda et al., 2012). The differences maybe due to different recording parameters (for example, holding potential, ion composition of pipette solution, and the usage of high-pass and low-pass filters), and the specific of recorded motoneurons (all the recorded cells in our study are identified as motoneurons). Although it was observed that MOR agonists, such as EM-2 or DAMGO, have inhibitory effects through binding to MORs expressed on spinal ventral motoneurons, serious locomotion inhibition rarely happens in clinic. The reasons might be as follows: (1) the dosage of EM or DAMGO is higher and it directly acts on motoneurons in patch-clamp recording; (2) some MOR-mediated suppressive effects on inhibitory neurotransmission counteract the suppressive effects on excitatory signal transduction.

Opioids also have excitatory effects in some central nervous system regions (Zieglgansberger et al., 1979; Piros et al., 1996). It has been demonstrated that intrathecal administration of both high- and low-dose opioids, could result in seizures in some cases (Kronenberg et al., 1998; Iff et al., 2012; Woodward et al., 2017). In addition, DAMGO decreased the frequency of sIPSC, suggesting that the opioids-induced presynaptic reduction of inhibitory neurotransmitter release might mediate the disinhibition of motoneurons (Honda et al., 2012). In this respect, it is reasonable to assume that opioids may induce muscle rigidity (Benthuysen et al., 1986) or spasticity (Kakinohana et al., 2003, 2006) via binding to the MORs, which might be attributable to the spinal effects of MOR-binding opioids, such as EM-2.

Although animal experiments showed that EMs had many edges in pain relief and other functions, the limitations still hampered the clinical application nowadays. Therefore, in recent years, the modification of EM has become a research focus. Fortunately, some novel peptides based on EM structure with high efficiency and extremely attenuated undesired effects (including motor impairment) have been developed (Zadina et al., 2016). In light of this, the application of EM analogs in clinic is very promising.

## Conclusion

The results of the present study suggest that morphine or other MOR agonists, such as EM-2, used at relevant dosages for antinociception in the spinal dorsal horn are also able to inhibit motoneuron activities though activating MORs located in the spinal ventral horn. The inhibitory effects of EM-2 on motoneurons in lamina IX at the spinal cord level may explain morphine-induced motion abnormality in clinic. The present research provides direct evidence for the side effects of EM-2 on locomotion. It has potential application value for preventing motor impairment induced by morphine and other opioids usage.

## Author Contributions

YL and HL initiated the project and design the present work. ZW, BF, YC, and TC performed the retrograde tracing and electrophysiological recordings. YL, HZ, TZ, and YD carried out the electron microscopy. ZW, YC, TC, YL, YB, HZ, and YD also contributed to preparation of figures, analysis and interpretation of data for this work. ZW and YC wrote the draft for the manuscript. YL and HL revised and edited the manuscript and approved the final vision of the manuscript.

## Conflict of Interest Statement

The authors declare that the research was conducted in the absence of any commercial or financial relationships that could be construed as a potential conflict of interest.
